# *QuickStats*: Percentage* of Adults Aged ≥18 Years Who Have Seen or Talked to a Doctor or Other Health Care Professional About Their Own Health in the Past 12 Months,^†^ by Sex and Age Group — National Health Interview Survey,^§^ United States, 2015

**DOI:** 10.15585/mmwr.mm6602a12

**Published:** 2017-01-20

**Figure:**
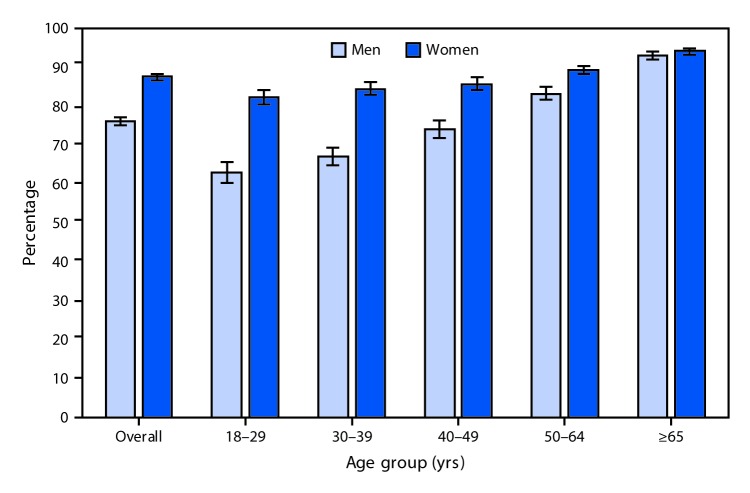
In 2015, women aged ≥18 years were more likely than men, overall and for each age group except those aged ≥65 years, to have seen or talked to a doctor or other health professional about their own health in the past 12 months. For both sexes, visits to a doctor or other health care professional increased with age, from 63.1% among men aged 18–29 years to 93.2% among men aged ≥65 years and from 82.4% among women aged 18–29 years to 94.3% among women ≥65 years.

**Source:** National Health Interview Survey, 2015. https://www.cdc.gov/nchs/nhis/index.htm.

